# Effect of Ultra-High Pressure on the Extraction of the Free, Esterified, and Bound Phenolics from *Dendrobium fimbriatum* Hook: Chemical Constituents and Antioxidant Ability

**DOI:** 10.3390/molecules30081836

**Published:** 2025-04-19

**Authors:** Qinge Su, Junbo Hu, Huimin Cui, Fangyuan Zheng, Yaping Liu, Zhengxuan Wang, Guiguang Cheng

**Affiliations:** 1 Faculty of Food Science and Engineering, Kunming University of Science and Technology, Kunming 650500, China; cjxhyyl@126.com (Q.S.); 18008899640@163.com (J.H.); 15368408779@163.com (H.C.); zfy199836@163.com (F.Z.); liuyaping@kust.edu.cn (Y.L.); 2 Yunnan International Joint Laboratory of Green Food Processing, Kunming 650500, China

**Keywords:** *Dendrobium fimbriatum* Hook, ultra-high pressure, UHPLC-OE-MS, phenolic fractions extraction, HepG2 oxidative stress model, Nrf2/Keap1 pathway activation

## Abstract

This study explores the antioxidant activity and antioxidant mechanism of phenolic compounds (including free (FP), esterified (EP), and bound phenolic (BP)) from *Dendrobium fimbriatum* Hook (DFH) stems, before and after ultra-high pressure (UHP) treatment. A total of 374 compounds were identified, with 149 showing significant differences in DFH phenolic extracts before and after UHP treatment. UHP treatment significantly increased the total phenolic content (TPC) and total flavonoid content (TFC) and enhanced antioxidant activity in vitro. Particularly, the UEP-DFH, IC_50_ values in ABTS and DPPH were reduced by 49.6% and 64.1%, respectively. In H_2_O_2_-treated HepG2 cells, the extracts demonstrated significant cytoprotective effects, including increased cell viability, ROS reduction, and enhanced GSH levels by 17.8% (UFP-DFH) and 12.5% (UEP-DFH). The activities of GS, GCL, GR, GSH-Px, SOD, CAT, NQO1, and HO-1 were also elevated in UHP-treated extracts. DAPI staining indicated that the extracts promoted nuclear Nrf2 expression, particularly UFP-DFH and UEP-DFH. Molecular docking indicated that vanillic acid could competitively bind to the Keap1-Kelch domain, facilitating activation of the antioxidant pathway. Overall, UHP treatment enhanced both extraction efficiency and antioxidant activity, making it a promising method for improving the bioactivity of DFH in the food and functional food industries.

## 1. Introduction

Phenolic compounds, as ubiquitous secondary metabolites in plants, fruits, and vegetables, play a crucial role in human health protection through their bioactive properties [1,2]. These compounds have significant therapeutic potential in mitigating chronic diseases, particularly cardiovascular diseases and hypertension, due to their potent antioxidant capacity [3]. Generally, phenolics exist in free (FP), esterified (EP), and bound forms (BP) in plant cells [4]. Despite the fact that they have nutritional and pharmacological potential, recent studies have predominantly focused on soluble FP and EP compounds, and research on bound phenolics is scarce, potentially limiting our understanding of their full biological activity.

Reactive oxygen species (ROS)-induced oxidative stress is a pivotal driver in the pathogenesis of chronic diseases, including neurodegenerative disorders, cardiovascular dysfunction, and metabolic syndromes [5]. Notably, excessive ROS generation and subsequent malondialdehyde (MDA) formation promote inflammatory cascades and programmed cell death via oxidative modifications of essential biological macromolecules, particularly nucleic acids, membrane lipids, and functional proteins [6]. Consequently, targeting ROS-scavenging pathways have emerged as a pivotal research focus of current research. Contemporary pharmacological studies demonstrate that phytochemicals exhibit potent ROS scavenging capacity through modulation of key oxidative stress markers. For example, curcumin analogs could significantly upregulate the expressions of nuclear factor erythroid-2-related factor 2 (Nrf2) and heme oxygenase-1(HO-1) to activate the Nrf2/HO-1 pathway, thereby enhancing cellular viability and mitigating oxidative stress [7]. Similarly, lignin–carbohydrate complexes (LCCs) could prevent the reduction of antioxidant enzyme activity in oxidative stress and effectively scavenge the endogenous ROS [8]. This scientific evidence underscores the importance of discovering novel natural antioxidants as potential therapeutic agents for managing oxidative stress-associated diseases.

Ultra-high pressure (UHP) treatment, an advanced non-thermal processing method that operates between 100 and 1000 MPa, has gained recognition as an effective technique for altering the physicochemical characteristics of plant-based materials [9]. The fundamental mechanism of UHP treatment involves the disruption of three-dimensional molecular structures and alteration of cellular organization through the breakdown of intermolecular bonds, particularly hydrogen bonds and hydrophobic interactions [10]. These structural modifications significantly enhance the liberation of phenolic compounds from the cellular matrix, thereby improving their bioavailability and biological activity [11]. Recent research showed the efficacy of UHP treatment in enhancing the extraction yield of various bioactive substances, including ginsenosides, flavonoids, and phenolic compounds [12,13,14]. Moreover, UHP treatment could significantly improve intracellular bioactivities of *Lyonia ovalifolia* compared with microwave, subcritical, and ultrasonic treatments [15]. These results collectively highlight the versatility and effectiveness of UHP treatment in the field of bioactive compound extraction.

*Dendrobium fimbriata* Hook (DFH) is a rare and valuable edible medicinal orchid endemic in China, which holds significant importance in traditional Chinese medicine (TCM) and is predominantly distributed in Yunnan Province [16]. This species has been traditionally applied in functionals foods due to its significant biological activities [17]. Our previous study reported that liquid–liquid extraction and macroporous adsorption resin separation techniques could effectively enrich the bioactive compounds, and some polar fractions exhibited stronger antioxidant ability than the methanol extract [18]. However, this previous study focused on the free and esterified phenolics, while the bound phenolic components of DFH were not effectively extracted, and its antioxidant effect was ignored.

As an advanced processing technology, UHP can enhance the anti-oxidative stress effect of natural antioxidants while providing theoretical support for expanding DFH applications to more fields. Currently, the changes in structural diversity and biological activity of FP, EP, and BP form DFH before and after UHP treatment remain unstudied. Therefore, in this study, FP, EP and BP were obtained from the dried stem of DFH before and after UHP treatment and were analyzed by the UHPLC coupled with Oregano Electronpray Mass Spectrometry (UHPLC-OE-MS). Furthermore, the antioxidant ability including 2,2-Diphenyl-1-picrylhydrazyl (DPPH), 2,2′-Azino-bis (3-ethylbenzothiazoline-6-sulfonic acid) (ABTS), and ferric-reducing antioxidant power (FRAP) assays, and cytoprotective effect on H_2_O_2_-induced HepG2 cell were also performed.

## 2. Results

### 2.1. UHPLC-OE-MS Analysis of Chemical Constituents in DFH Extracts

**Figure 1:**
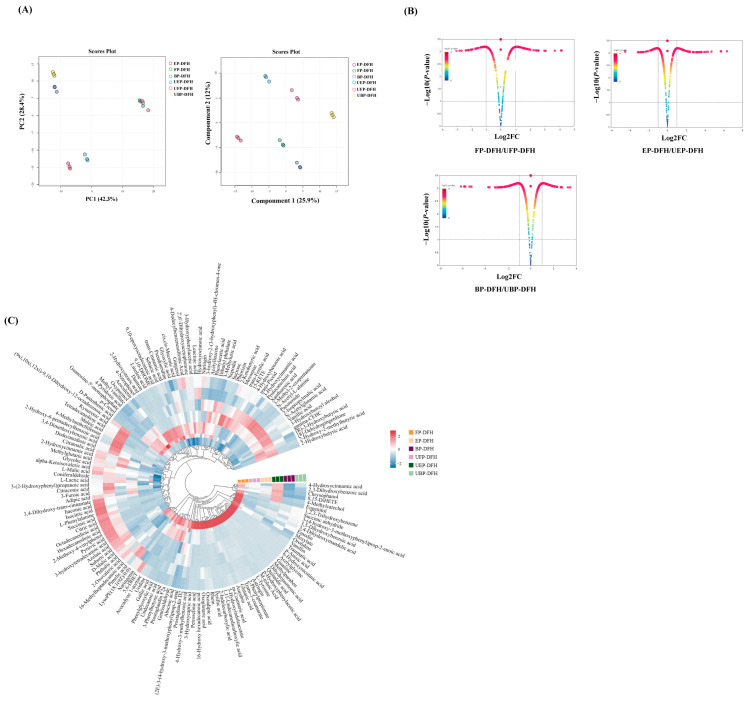
(**A**) PCA analysis and PLS-DA analysis of metabolites in various DFH extracts; (**B**) volcanic maps of various DFH extracts; (**C**) heatmap of clustering of various DFH extracts.

In order to explain the effect of UHP on chemical constituents in DFH extracts, systematic research of the phenolics on free, esterified, and bound forms extracted from DFH before and after UHP treatment were performed by untargeted metabolomic analysis. Through the detailed analysis of accurate molecular weight and ms^2^ data, a total of 374 chemical compounds were identified from different DFH extracts in the negative mode, including 28 phenolic acids, 25 flavonoids, 25 hydroxy acids, 25 phenols, 22 fatty acids, 20 dicarboxylic acids, 15 amino acid derivatives, and so on (Table S1). The differences of these chemical compounds in various DFH extracts were further investigated using the variable importance in projection (VIP) values in the partial least square-discriminant analysis (PLS-DA) model analyzed by SIMCA-P 13.0 software (Sartorius Stedim Data Analytics, Umeå, Sweden). The results revealed that the parallelism of sample detection was good and there was a good separation efficacy between each fraction (Figure 1A). The volcanic map result (Figure 1B) demonstrated that UHP treatment could obviously affect the chemical constituents of FP, EP, and BP in various DFH extracts. It showed that in FP-DFH/UFP-DFH, the numbers of upregulated and downregulated compounds were comparable, while EP-DFH/UEP-DFH exhibited predominantly upregulated compounds, and BP-DFH/UBP-DFH showed a majority of downregulated compounds. For screening the differential chemical compounds that contributed most to differentiating the six DFH extracts, the screening condition of VIP > 1.25 and *p* < 0.05 was used to select differential compounds, of which there were 149 compounds differentially distributed in these six DFH extracts (Table S2 and Figure 1C). The cluster heatmap of DFH extracts in Figure 1C revealed the effect of UHP on the chemical composition of FP, EP, and BP across different DFH extracts. FP-DFH showed the highest number of upregulated compound types, while UEP-DFH contained significantly more upregulated compounds than EP-DFH. Furthermore, different treatments had an effect on the compounds in different groups. These results demonstrated that UHP treatment can significantly alter the chemical composition of FP, EP, and BP in DFH.

**Figure 2:**
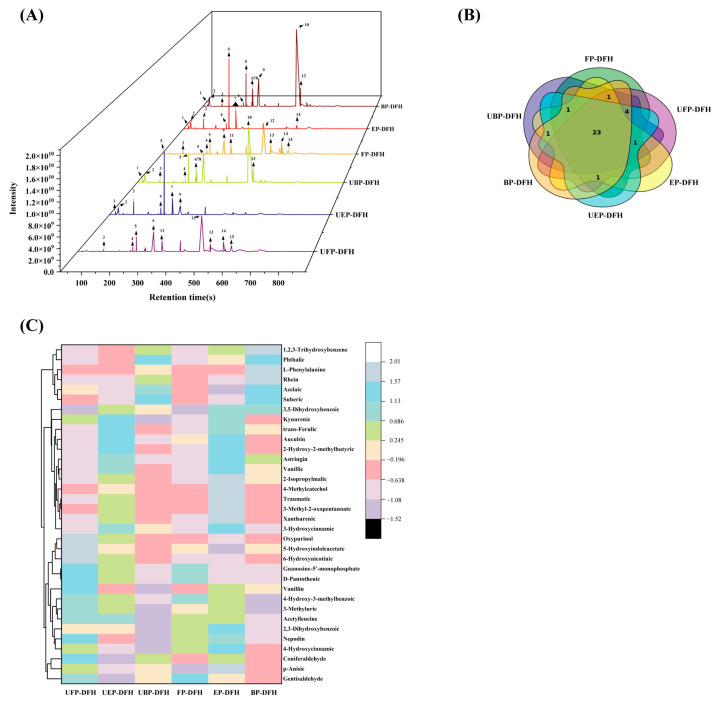
(**A**) The total ion chromatograms of main compounds from various DFH extracts in negative mode; (**B**) Venn diagram analysis of various DFH extracts; (**C**) cluster heatmap of the main compounds in various DFH extracts.

For a better understanding of the main chemical compounds, 32 chemical constituents with higher contents were detected in the total ion chromatograms of FP-DFH, EP-DFH, BP-DFH, UFP-DFH, UEP-DFH, and UBP-DFH (Figure 2A). Obviously, the peak numbers and heights of detected compounds exhibited a significant difference, indicating that UHP treatment could remarkably influence the chemical composition of DFH. For instance, compared to FP-DFH, the peak heights of compounds **9** and **11** in UFP-DFH increased significantly. Meanwhile, the peak height of compound **10** in UEP-DFH decreased markedly compared to EP-DFH. Additionally, the Venn diagram (Figure 2B) showed that the compounds detected in these six DFH extracts also exhibited considerable similarity (23 common chemical compounds) as well as some differences, where 32 compounds were found in FP-DFH, UEP-DFH, BP-DFH, and UBP-DFH, while 30 and 28 compounds were found in UFP-DFH and EP-DFH, respectively. To intuitively determine the difference between various DFH extracts, these main compounds were analyzed by a heatmap (Figure 2C), and the results are summarized in Table 1. The results revealed that 2-hydroxy-2-methylbutyric acid, 2-isopropylmalic acid, vanillic acid, and azelaic acid exhibited significantly higher relative abundances compared to other compounds. Notably, esterified phenolics (e.g., 2-Hydroxy-2-methylbutyric acid, 2-Isopropylmalic acid, and vanillic acid) showed higher relative abundances than their free and bound phenolics, suggesting that UHP treatment not only released additional compounds but also preferentially enriched the esterified phenolics. In contrast, the chemical constituents in the UBP-DFH were significantly decreased than BP-DFH. This may be the reason that UHP treatment might destroy the structure of BP, which was released as free or esterified phenolics. The results demonstrated that UHP treatment could significantly change the extraction yield and chemical compounds in DFH extracts.

### 2.2. Determinations of Total Polyphenol Content (TPC) and Total Flavonoid Content (TFC) Levels in Different Forms of Phenolics in DFH

**Figure 3:**
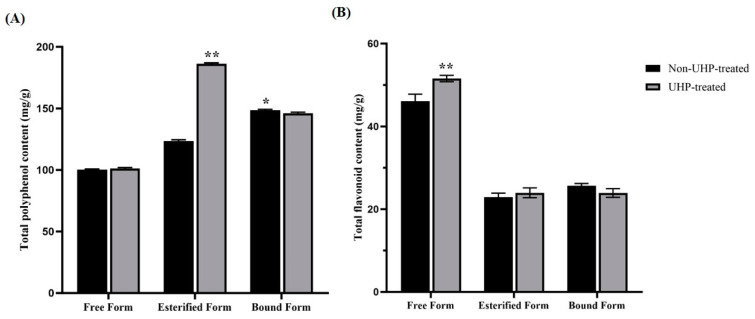
Contents of phenolic content (TPC) (**A**) and total flavone content (TFC) (**B**) in various DFH extracts before and after UHP treatment. “*” indicates *p* < 0.05, “**” indicates *p* < 0.01.

UHPLC-OE-MS analysis exhibited that there was a significant difference in chemical compounds in these six DFH extracts, and the phenolics and flavonoids were the main chemical compositions. Furthermore, the difference in TPC and TFC values of various phenolic forms were determined. As shown in Figure 3A,B, UHP treatment significantly enhanced EP content to 183.64 ± 3.34 mg gallic acid equivalent (GAE/g extract), representing a 1.53-fold increase versus the non-treated group (*p* < 0.01). As for TFC, the UFP-DFH group contained the highest TFC (51.63 ± 0.87 mg rutin equivalent (RE/g extract)), followed by the untreated FP-DFH group (42.43 ± 0.44 mg RE/g extract). Although the TPC level in the UFP-DFH group and the TFC level in UEP-DFH group increased compared to the untreated groups, there were no significant differences (*p* > 0.05). In contrast, the TPC and TFC levels in both UBF-DFH groups decreased in comparison with the BF groups. The results indicated that UHP treatment could increase the accumulation of free and esterified phenolic compounds in comparison to bound phenols.

### 2.3. In Vitro Antioxidant Activity in Different DFH Extracts

**Figure 4:**
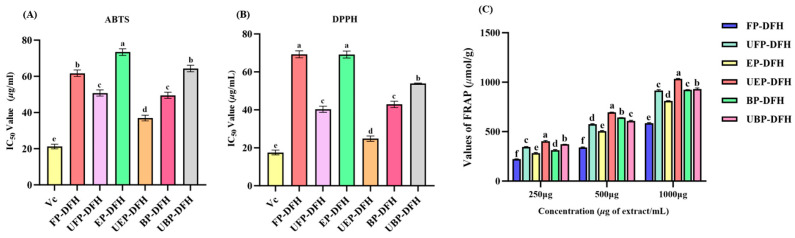
The IC_50_ values of ABTS (**A**) and DPPH (**B**), and the FRAP values (**C**) of various DFH extracts before and after UHP treatment. Different letters in each column indicate significant differences (*p* < 0.05) among different treatments.

ABTS radical and DPPH radical scavenging abilities and FRAP values are generally used for estimating in vitro antioxidant ability of bioactive compounds. As shown in Figure 4, all phenolic fractions from DFH exhibited significant antioxidant activity with IC_50_ values < 100 μg/mL, indicative of their potential applications as natural antioxidants. Figure 4 reveals that UHP treatment significantly enhanced the radical scavenging capabilities of most fractions: the IC_50_ values of ABTS (Figure 4A) in UFP-DFH and UEP-DFH groups obviously decreased by 16.9% and 49.6% (*p* < 0.01) and the IC_50_ values of DPPH (Figure 4B) in UFP-DFH and UEP-DFH groups also significantly decreased by 41.8% and 64.1% (*p* < 0.01), respectively, in comparison with the untreated DFH groups. Similar results were also found in the FRAP values (Figure 4C), where UHP treatment significantly increased FRAP values in the UFP-DFH and UEP-DFH groups. However, this trend did not hold for the UBP-DFH group, where IC_50_ values of ABTS and DPPH significantly increased, accompanied by a decrease in FRAP values.

Further analysis revealed significant negative correlations between TPC/TFC levels and IC_50_ values of ABTS and DPPH, with R^2^-values as low as −0.875 (for TPC and ABTS), −0.892 (for TFC and ABTS), −0.881 (for TPC and DPPH), and −0.902 (for TFC and DPPH). Conversely, a strong positive correlation was observed between these TPC/TFC indices and the FRAP values (r = 0.913 for TPC and FRAP value; r = 0.894 for TFC and FRAP value), suggesting potential correlations between polyphenolic content distribution and antioxidant ability. The differential effects of UHP treatment on these bioactive compounds indicate structural modifications that enhanced the extractability and bioavailability of free and esterified phenolic groups, potentially due to increased water solubility or altered molecular interactions.

### 2.4. Cytoprotective Activity in Different DFH Extracts in H_2_O_2_-Treated HepG2 Cells

#### 2.4.1. The Influence of Different DFH Extracts on H_2_O_2_-Induced HepG2 Cell Viability and Morphology

**Figure 5:**
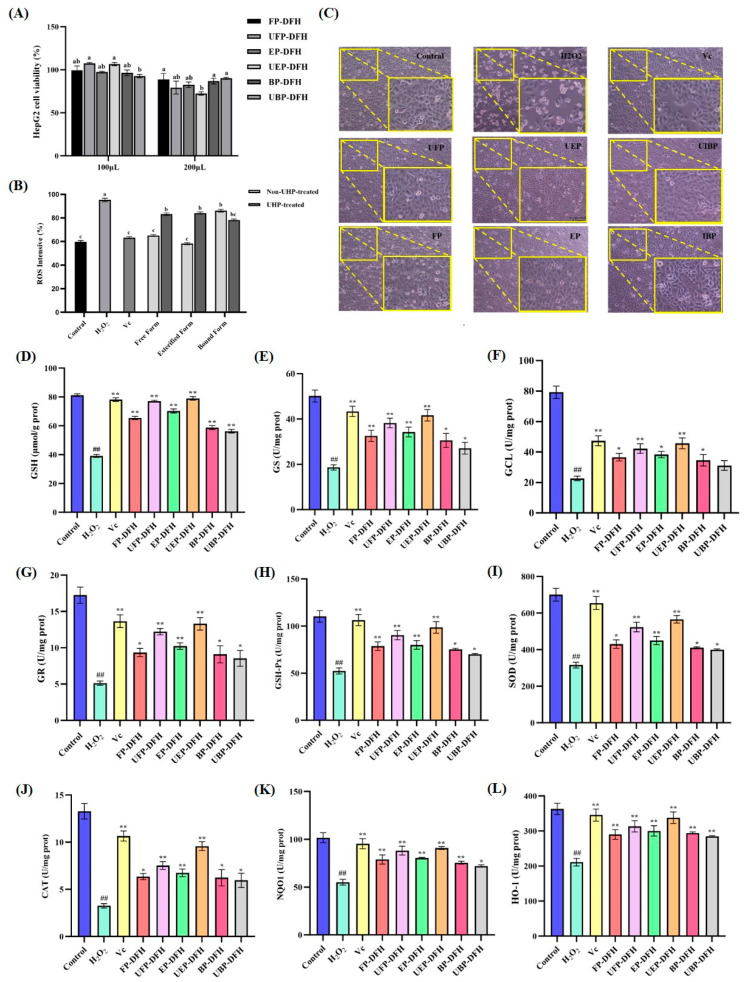
The effects of various DFH extracts treatment on the viability, morphology, ROS accumulation, and glutathione (GSH) synthesis, and metabolism in H_2_O_2_-induced HepG2 cells. (**A**) The viability of HepG2 cells; (**B**) intracellular ROS content; (**C**) the morphology of HepG2 cells; (**D**) GSH content; the activity of (**E**) glutathione synthetase (GS); (**F**) glutamate-cysteine ligase (GCL); (**G**) glutathione reductase (GR); (**H**) glutathione peroxidase (GSH-Px); (**I**) superoxide dismutase (SOD); (**J**) catalase (CAT); (**K**) NAD(P)H quinone oxidoreductase 1 (NQO1); and (**L**) heme oxygenase-1 (HO-1). All experimental data were expressed as “Mean ± SD” (*n* = 3), “*” represents *p* < 0.05 between M group and sample groups, “**” indicates *p* < 0.01 between M group and sample groups, “##” represents significant differences among M group and sample groups, blank group and positive group (*p* < 0.01).

The cytotoxic effects of DFH extracts (FP-DFH, EP-DFH, BP-DFH, UFP-DFH, UEP-DFH, and UBP-DFH) on HepG2 cells were assessed using the methylthiazol-2-yl-2,5-diphenyl tetrazolium bromide (MTT) assay. As illustrated in Figure 5A, the cell viability at 100 μg/mL was significantly higher than at 200 μg/mL. After the co-incubation with six DFH extracts at 100 μg/mL, the cell survival rates exceeded 90%, confirming the absence of cytotoxicity and validating this concentration for further experiments.

Cell morphology analysis provided further insights into the protective effects of DFH extracts [19]. As shown in Figure 5C, HepG2 cells exhibited a spindle-like morphology with distinct boundaries. However, H_2_O_2_ exposure caused a marked reduction in cell number, accompanied by rounding, fragmentation, and debris formation. In contrast, DFH-treated groups cells maintained near-normal morphology, with minimal rounding and superior protection compared to vitamin C (Vc)-treated cells. Simultaneously, the diagram indicated that UEP-DFH exhibited the best cell protection effect, ensuring both cell viability and morphological integrity. Notably, the protective effects of free, esterified, and bound phenolics in DFH groups on cells before and after UHP treatment were compared. It is evident that the protective efficacy of UBP-DFH was less optimal, while other components subjected to UHP treatment demonstrated superior performance compared to their untreated counterparts.

#### 2.4.2. Effect of DFH Phenolic Fractions on ROS Production in H_2_O_2_-Induced HepG2 Cells

Excessive ROS production disrupted cellular antioxidant defenses, leading to oxidative damage [20]. To evaluate the antioxidative potential of DFH extracts (FP-DFH, EP-DFH, BP-DFH, UFP-DFH, UEP-DFH, and UBP-DFH) against H_2_O_2_-induced oxidative stress, ROS levels in HepG2 cells were quantified in Figure 5B. Compared to the control group, H_2_O_2_ exposure increased intracellular ROS by 1.59-fold (*p* < 0.01), while treatment with DFH extracts significantly reduced ROS levels. Notably, UFP-DFH and UEP-DFH groups exhibited lower ROS levels than untreated DFH extracts, where UEP-DFH showed the best antioxidant activity, even superior to the Vc. These findings demonstrated that DFH extracts effectively mitigated ROS accumulation, and UHP treatment could significantly improve the antioxidant capacity of DFH.

#### 2.4.3. Effect of DFH Phenolic Fractions on Glutathione (GSH) Synthesis and Metabolism in H_2_O_2_-Induced HepG2 Cells

GSH is a critical intracellular antioxidant and serves as a key indicator of cellular antioxidant capacity [21]. Figure 5D illustrates that H_2_O_2_ treatment significantly reduced GSH levels by 2.08-fold compared to the control group (*p* < 0.01). Notably, all DFH extracts (FP-DFH, EP-DFH, BP-DFH, UFP-DFH, UEP-DFH, and UBP-DFH) significantly increased the GSH levels in HepG2 cells, enhancing their antioxidant capacity. Compared to the DFH groups without UHP treatment, the GSH levels in UFP-DFH and UEP-DFH increased by 17.8% and 12.5%, respectively, whereas the GSH level in UBP-DFH decreased by 4.3%. This change in GSH levels may be closely associated with the reduction in total phenolic flavonoids observed in UBP-DFH.

The synthesis and metabolism of GSH are regulated by key enzymes of glutathione synthetase (GS) and glutamate-cysteine ligase (GCL), while glutathione reductase (GR) and glutathione peroxidase (GSH-Px) mediate its regeneration and utilization for antioxidant ability [22,23]. Analysis of enzyme activities in HepG2 cells revealed that DFH treatment significantly elevated GS (Figure 5E), GCL (Figure 5F), GR (Figure 5G), and GSH-Px (Figure 5H) activities compared to the model group. Notably, UHP-treated UFP-DFH and UEP-DFH groups exhibited higher enzyme activities than untreated DFH extracts, aligning with the observed increase in GSH content. Among these, UEP-DFH demonstrated the most pronounced enhancement in GSH synthesis and metabolism, with similar efficacy to the positive control Vc. These enzymes activities were all decreased in UBP-DFH group, which was consistent with its GSH results. These results underscore the ability of UHP treatment to amplify the antioxidant capacity of free and esterified phenolics.

#### 2.4.4. Effect of DFH Phenolic Fractions on Activities of Antioxidant Enzymes in H_2_O_2_-Induced HepG2 Cells

Superoxide dismutase (SOD), catalase (CAT), NAD(P)H quinone oxidoreductase 1 (NQO1), and heme oxygenase-1 (HO-1) are crucial markers for evaluating the potential antioxidant and cytoprotective effects of bioactive compounds, as they play pivotal roles in the cellular antioxidant defense system [24]. The results (Figure 5I–L) demonstrated that compared with the H_2_O_2_-induced model group, all DFH extracts groups significantly increased the activity of SOD (Figure 5I), CAT (Figure 5J), NQO1 (Figure 5K), and HO-1 (Figure 5L) in HepG2 cells. These findings highlight the ability of DFH extracts to counteract hydrogen peroxide-induced oxidative damage and protect cells. Notably, the UFP-DFH and UEP-DFH groups exhibited the most pronounced increases in SOD, CAT, NQO1, and HO-1 activities, highlighting their superior antioxidant capacity. These results are consistent with previous experimental observations.

### 2.5. DAPI Staining Results and Molecular Docking Analysis

**Figure 6:**
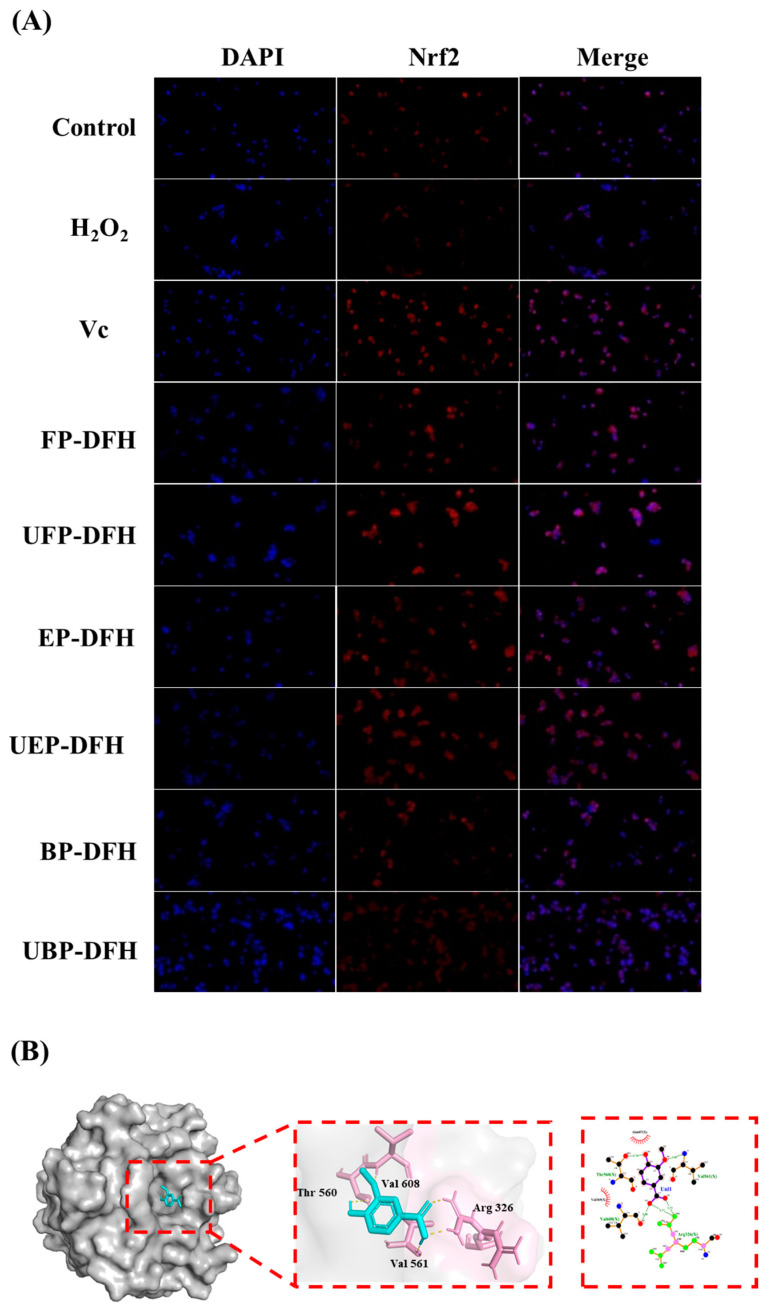
(**A**) Fluorescence microscopy after DAPI staining showed the expression of NRF2 in HepG2 cells induced by hydrogen peroxide (Blue indicates the localization and distribution of the nucleus, and red indicates the nuclear expression of Nfr2). (**B**) Molecular docking of Keap1-Kelch and the native ligand Nrf2 is shown as 3D diagram and 2D diagram.

The Nrf2 signaling pathway plays an indispensable role in enhancing cellular antioxidant defenses and protecting cells from oxidative stress-induced injury [25]. Nrf2 nuclear localization and distribution can be clearly visualized by DAPI staining, especially the nuclear expression of Nrf2 [26]. As shown in Figure 6A, DFH extracts significantly enhanced Nrf2 nuclear translocation, with the FP-DFH, EP-DFH, UFP-DFH, and UEP-DFH groups exhibiting particularly pronounced effects. These results suggest that DFH extracts could activate Nrf2 to upregulate antioxidant enzyme activity in H_2_O_2_-induced HepG2 cells.

Molecular docking was employed to explore the binding interactions between vanillic acid and the Keap1-Kelch domains [27]. As illustrated in Figure 6B, Nrf2 formed hydrogen bonds with Keap1-Kelch residues (Thr560, Val561, Val608, Arg326) and interacted with Thr560 and Val608 via salt bridges/attractive interactions involving Val369 and Ala607. These findings indicate that vanillic acid competitively occupied the Nrf2 binding site in the Keap1-Kelch domain, inhibiting Keap1-Nrf2 interactions, which promoted Nrf2 nuclear accumulation under oxidative stress, reducing cellular damage and enhancing protection.

### 2.6. Principal Component and Relationship Analysis

**Figure 7:**
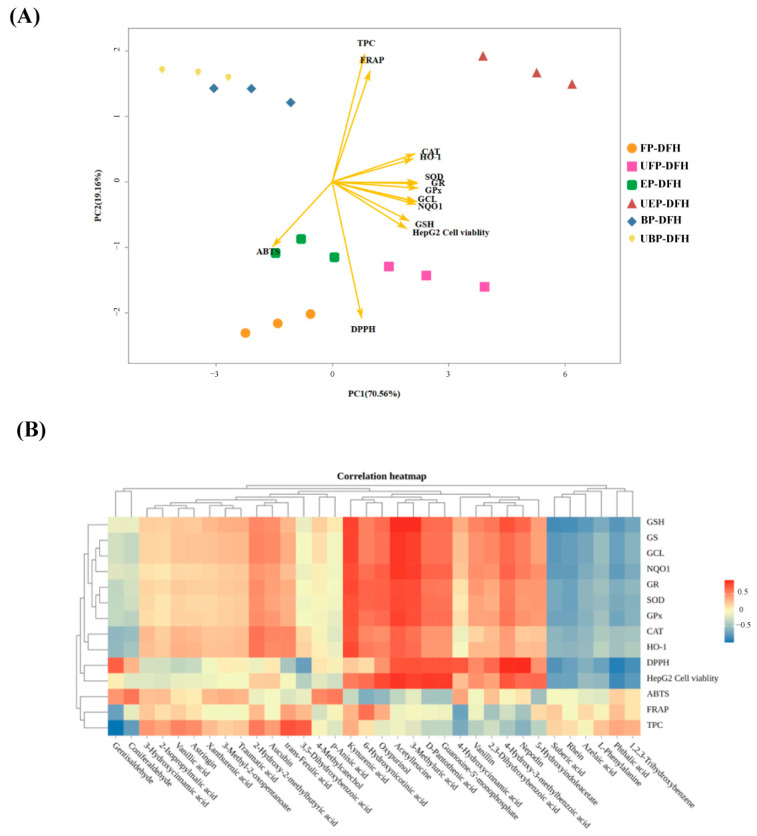
(**A**) The results of principal component analysis (PCA). (**B**) The cluster heatmap of the relationship between the main compounds in various DFH extracts and determined biological indexes.

Principal component analysis (PCA) was performed to comprehensively evaluate the data of this study. As shown in Figure 7A, PC1 and PC2 explained 70.56% and 19.61% of the total variance, respectively. Except for UBP-DFH, the UHP-treated DFH groups (UFP-DFH and UEP-DFH) were predominantly distributed on the right side of the PCA plot, while non-UHP-treated groups (FP-DFH, EP-DFH, and BP-DFH) clustered on the left. This spatial separation suggested that UHP treatment significantly modifies the composition and functionality of DFH components, particularly in UFP-DFH and UEP-DFH. Furthermore, DFH extracts showed strong positive correlations with antioxidant enzymes (SOD, CAT, NQO1, and HO-1), GSH synthesis and metabolism enzymes (GS, GCL, GR, and GSH-Px), and Nrf2 protein activity, with UFP-DFH and UEP-DFH demonstrating the most pronounced antioxidant effects.

The heatmap analysis (Figure 7B) identified 34 metabolites strongly correlated with antioxidant indexes, with distinct clustering patterns reflecting their differential associations. Notably, metabolites such as kynurenic acid, acetylleucine, and 3-methyluric acid exhibited high positive correlations with intracellular antioxidant enzymes (SOD, CAT, NQO1, and HO-1), GSH synthesis and metabolism enzymes (GS, GCL, GR, and GSH-Px), and cellular viability (HepG2). This suggested that these metabolites may synergistically enhance cellular antioxidant defense systems by modulating enzymatic activity or redox homeostasis, consistent with the results of PCA analysis.

In contrast, ABTS, FRAP, and TPC showed weaker correlations with the aforementioned metabolites. Interestingly, trans-ferulic acid emerged as the metabolite most strongly associated with TPC, aligning with its well-characterized role as a phenolic compound with high hydroxyl group availability for redox reactions. This divergence may reflect that diverse DFH metabolites have differences in antioxidant mechanisms and highlight the close relationship between these metabolites and their robust antioxidant capacity, consistent with previous research by Lugo-Huitrón et al. [28].

## 3. Discussion

The advancement of environmentally friendly and efficient extraction methods plays a crucial role in enhancing both the yield and the bioactive chemical components of edible plant-derived resources [29]. Compared to FP and EP, BP is generally ignored because it is tightly bonded with polysaccharides or proteins through covalent bonds in the cell wall matrix of plants, resulting in lower extraction efficacy. Therefore, this research compared the chemical constituents and biological activities of phenolic fractions (FP, EP, and BP) in DFH before and after UHP treatment.

The differential effects of UHP treatment on polyphenolic fractions, particularly the preferential accumulation of FP and EP over BP, can be attributed to its mechanochemical actions on plant cellular structures and altered cellular dynamics under pressure-induced stress [30]. UHP-enhanced FP/EP extractability primarily stems from the following: (1) cellular structural disruption enabling solvent penetration, and (2) phenolic liberation via altered lipophilic conformations and disrupted protein–solvent bonds, promoting bioactive migration and stabilization through membrane permeabilization mechanisms.

Conversely, UHP treatment reduces BP through partial degradation of covalent linkages between phenolics and cell wall components (e.g., cellulose/hemicellulose) and pressure-induced conformational changes in structural proteins that alter phenolic binding sites [31]. Our previous study [32] revealed that UHP treatment significantly enhanced extraction efficiency, leading to elevated levels of TPC and TFC in Quezui tea. In this study, similar results were also found where UHP treatment increased the TPC and TFC levels in DFH extract, especially in FP-DFH and EP-DFH groups, but reduced in the BP-DFH group. These molecular rearrangements not only explain the UHP-induced phenolic migration but also highlight the potential for developing novel natural antioxidant compounds using supercritical processing techniques.

ROS plays a dual role as signaling molecules and mediators of oxidative stress. While low ROS levels regulate physiological processes, excessive ROS induces oxidative damage [33]. For instance, in Lu et al.’s study, excessive ROS accumulation was found to cause liver damage, while the ethyl acetate extract of *V. ciliata* alleviated oxidative stress by activating the AMPK/p62/Nrf2 pathway, thereby mitigating oxidative stress-induced liver disease [34]. This aligns with our findings on DFH extracts, particularly the UHP-treated fraction, which effectively scavenges ROS and alleviates oxidative stress-induced damage. In this study, DFH extracts significantly reduced intracellular ROS levels, with UHP-treated free and esterified phenolics showing the most pronounced effects. Antioxidant activity was further evaluated using ABTS, DPPH, and FRAP assays [35]. All DFH extracts demonstrated strong in vitro antioxidant activity, with UHP-treated UEP-DFH and UFP-DFH exhibiting superior ABTS and DPPH radical scavenging capacities and enhanced FRAP values compared to untreated samples (EP-DFH and FP-DFH). Similarly, studies demonstrating enhanced antioxidant ability following UHP treatment have been reported in other phenol-rich plants. For example, UHP treatment of FP, EP, and BP from *Anneslea fragrans* Wall revealed that UHP-treated samples exhibited the strongest inhibition of ROS production [14]. Notably, UHP treatment improved the antioxidant activity of free and esterified phenolics but not bound phenolics, likely due to structural and compositional differences.

GSH, the predominant non-enzymatic antioxidant, serves as the secondary defense mechanism within the endogenous antioxidant network [36]. Its biosynthesis is tightly controlled through the sequential actions of GCL (the rate-limiting enzyme) and GS [37]. GSH collaborates with GSH-Px to neutralize the lipid peroxidation byproduct induced by ROS, including MDA [38]. The antioxidative capacity of GSH is sustained via a redox cycle of oxidized glutathione (GSSG), which is regenerated to GSH by GR catalyzation [39]. Consequently, augmenting GSH biosynthesis and redox cycling represents a critical strategy for counteracting oxidative stress. In this study, UHP treatments markedly enhanced the regulatory efficiency of FP and EP from DFH on GSH synthesis and metabolism in H_2_O_2-_induced HepG2 cells by upregulating the activities of key enzymes, including GCL, GS, GR, and GSH-Px.

Antioxidative enzymes, such as SOD, CAT, HO-1, and NQO1, are the first defense system in the body for scavenging ROS accumulation and reduced oxidative stress damage [40]. Studies have shown that ROS could be sequentially metabolized into water and O_2_ through the catalyzation of SOD and CAT. In parallel, HO-1 contributes to antioxidant defense by degrading heme into biliverdin, carbon monoxide, and free iron, among which biliverdin is further metabolized into bilirubin, a potent antioxidant that directly scavenges ROS [41]. Additionally, NQO1 alleviates oxidative stress via two-electron quinone-to-hydroquinone conversion, suppressing ROS formation. [42]. In our study, UHP treatments markedly enhanced these antioxidant enzyme activities of FP and EP from DFH in H_2_O_2-_induced HepG2 cells, highlighting the superior benefits of UHP treatment on improving antioxidant capacity of phenolic extracts.

Nrf2 serves as a central regulator of oxidative stress and redox balance. Normally, Nrf2 is combined with Kelch-like ECH-associated protein 1 (Keap1) with no activity. After stimulation by free radicals (especially ROS), Nrf2 releases from the Keap1/Nrf2 protein complex and immigrate to nucleus, which will then combine with antioxidant response element (ARE) to induce expression of GSH production and antioxidative enzymes (e.g., SOD, CAT, NQO1, and HO-1) [43]. Studies have demonstrated that Nrf2 signaling promotes GSH biosynthesis and metabolism by enhancing activities of GCL, GS, GR, GSH-Px, etc. [44]. Notably, epigallocatechin gallate (EGCG) can activate Nrf2 expression and function, effectively mitigating diabetes-induced renal oxidative damage, inflammation, fibrosis, and proteinuria [45]. This aligns with our research that UHP-treated DFH extracts enhanced Nrf2 nuclear translocation, suggesting a shared Nrf2 activation mechanism among plant-derived antioxidants. This Nrf2-mediated antioxidant network plays a critical role in suppressing cellular ROS generation. In the current study, the DAPI staining results showed UHP treatment significantly increased Nrf2 expression in nuclear in H_2_O_2_-induced HepG2 cells, indicating that antioxidative effects of DFH phenolic fractions are likely mediated through Nrf2 transcriptional activation.

To elucidate the mechanisms of Nrf2 activation, the chemical composition of DFH was systematically analyzed. UHP treatment modified the levels of several phenolic compounds, including vanillic acid, traumatic acid, and azelaic acid, many of which exhibit antioxidant properties [46,47]. As reported by Mentese et al. [48], vanillic acid could activate the Nrf2 pathway and reduce oxidative damage in cells, further supporting its role as a key bioactive compound in DFH extracts and contributing to oxidative stress mitigation. Therefore, vanillic acid was further selected for molecular docking experiments to verify its activation of Nrf2. The results confirmed its strong binding affinity to Nrf2, inhibiting Keap1-Nrf2 interactions and promoting Nrf2 pathway activation.

Conclusively, the chemical compositions and antioxidant activity of DFH extracts were significantly enhanced following UHP treatment. Principal component analysis (PCA) in this study revealed that the DFH extract exhibited a significant correlation with antioxidant activity, with its antioxidant activity being significantly enhanced following UHP treatment. It is worth noting that the IC_50_ of ABTS and DPPH in UFP-DFH decreased by 49.6% and 64.1%, respectively; increased intracellular GSH levels by 12.5%; and showed a significant correlation with antioxidant enzymes (e.g., SOD, CAT, HO-1, and NQO1). The results showed that UHP treatment significantly improved the antioxidant capacity of DFH extracts by promoting the GHS metabolism and synthesis of UFP-DFH and UEP-DFH and the activity of antioxidant enzymes, thereby enhancing the overall antioxidant activity. These findings not only clarify the regulation mechanism of antioxidant activity of different DFH extracts but also provided valuable insights into applications of UHP-treated DFH extracts in the development of functional foods and nutraceuticals with antioxidant potential.

## 4. Materials and Methods

### 4.1. Chemicals and Reagents

2,2′-azinobis (3-ethylbenzothiazoline-6-sulfonic acid) diammonium salt (ABTS, purity ≥ 98%), 1,1-diphenyl-2-picrylhydrazyl (DPPH, purity ≥ 95%), 3-(4,5-dimethyl-2-thiazolyl)-2,5-diphenyl-2-H-tetrazolium bromide (MTT, purity ≥ 97.5%), and 2′,7′-dichlorofluorescin diacetate (DCFH-DA, purity ≥ 97%) were obtained from Sigma-Aldrich (Shanghai, China). Fetal bovine serum (FBS), penicillin, streptomycin, and Dulbecco’s modified Eagle’s medium (DMEM) were sourced from Beijing Solaibao Technology Co., Ltd. (Beijing, China). The human hepatocellular carcinoma cell line (HepG2) was procured from the Kunming Cell Bank (Chinese Academy of Sciences, Kunming, China). Additionally, assay kits for superoxide dismutase (SOD), reactive oxygen species (ROS), glutathione (GSH), glutathione peroxidase (GSH-Px), and glutathione S-transferase (GST) were purchased from Nanjing Jiancheng Bioengineering Research Institute (Nanjing, China). Foline-phenol (purity ≥ 99%) and trypsin (≥2500 U/mg) were also acquired from Sigma-Aldrich (Shanghai, China).

### 4.2. Sample Collection and Preparation

The stems of DFH were collected from Pu’er City in Yunnan Province of China. The dried powder of DFH stems over 80-mesh sieve (20 g) was vacuum-sealed in packs and subjected to pretreatment using ultra-high pressure (UHP) equipment at 500 MPa for 10 min in water. The UHP system operated at a voltage of 380 V, and the temperature was consistently maintained at 25 °C. This experimental group was designated as the UHP treatment group.

### 4.3. Preparation of Free, Esterified, and Bound Phenolics

The extraction and optimization procedures were adapted from established methodologies [49]. Briefly, DFH stem powder (20 g) was firstly defatted by petroleum ether at a ratio of 1:10 (*w*/*v*) for 30 min by three times to remove non-polar lipids and pigments that could interfere with phenolic extraction and analysis. The defatted sample was then extracted using 70% methanol-acetone (1:1, *v*/*v*) aqueous solution by ultrasound-assisted extraction for 20 min. The supernatant obtained after centrifugation (4000 rpm, 20 min) was concentrated by rotary evaporation (RV 3 FLEX, IKA, Staufen, Germany) to yield the extract. For the fractionation of free phenolics (FP from DFH: FP-DFH; FP from UHP-DFH: UFP-DFH), the extract was extracted by ether and ethyl acetate (1:1, *v*/*v*) solution. The aqueous phase after FP extraction was hydrolyzed with 4 mol/L NaOH at room temperature for 4 h and adjusted to pH 2.0 by HCl. Esterified phenolics (EP of DFH: EP-DFH; EP of UHP-treated DFH: UEP-DFH) were then extracted using the same FP procedure, and then concentrated and lyophilized for further analysis. To obtain bound phenolics (BP of DFH: BP-DFH; UBP of UHP-treated DFH: UBP-DFH), the residual plant waste was re-suspended in 4 mol/L NaOH and hydrolyzed at room temperature for 4 h. The pH was adjusted to 2.0 by HCl, and the solution was centrifuged at 4000 m for 20 min. The supernatant was then extracted with ether and ethyl acetate solution five times, and then concentrated and lyophilized to obtain BP.

### 4.4. Chemical Constituent Analysis of DFH Extracts

The chemical composition of various DFH extracts (FP-DFH, EP-DFH, BP-DFH, UFP-DFH, UEP-DFH, and UBP-DFH) was characterized utilizing ultra-high performance liquid chromatography coupled with Oregano Electronpray Mass Spectrometry (UHPLC-OE-MS). The samples were dissolved in a methanol solution. The sample was analyzed using a Waters ACQUITY HSS T3 column (2.1 mm × 100 mm, 1.8 μm) maintained at 25 °C. The mobile phase consisted of (A) 5 mmol/L ammonium acetate and 5 mmol/L acetic acid in water and (B) acetonitrile. The gradient elution program was as follows: 0–0.7 min, 1% B; 0.7–11.8 min, 99% B; 11.8–12 min, 99–1% B; and 12–15 min, 1% B. The injection volume was 2 μL. Mass spectrometry analysis was conducted using a heated electrospray ionization source in negative ion mode, with spray voltages of +3.8 and −3.4 kV, sheath gas flow rate of 50 arb, capillary temperature of 320 °C, and auxiliary gas heater temperature of 350 °C. Data acquisition and processing were performed using Xcalibur 4.1 software (Thermo Scientific, Waltham, MA, USA).

### 4.5. Metabolic Diversity and Difference Analysis

Metabolomic analysis was performed using Compound Discoverer 3.3 software for raw data processing. Variable importance in projection (VIP) scores, principal component analysis (PCA), and partial least squares-discriminant analysis (PLS-DA) were conducted using SIMCA-P software. Volcano plots and hierarchical clustering heatmaps of DFH extracts (FP-DFH, EP-DFH, BP-DFH, UFP-DFH, UEP-DFH, and UBP-DFH) were generated using R (v4.2.3) programming.

### 4.6. Determination of Total Polyphenol (TPC) and Total Flavonoid (TFC) Content

#### 4.6.1. Determination of TPC Level

The total phenolic content (TPC) of each extract was determined according to the Folin–Ciocalteu method [50]. The samples were dissolved in 80% methanol to prepare a 0.2 mg/mL solution. A reaction mixture containing 0.1 mL Folin–Ciocalteu reagent, 0.2 mL sample solution, 0.3 mL Na_2_CO_3_ (20% *m*/*v*), and 1.2 mL ultrapure water were incubated at 70 °C for 10 min. The absorbance was measured at 765 nm using a spectrophotometer, and the TPC was expressed as milligrams of gallic acid equivalent (GAE) per gram of extract, calculated using a gallic acid standard curve (y = 110.63x + 0.0078, R^2^ = 0.999).

#### 4.6.2. Determination of TFC Level

The total flavonoid content (TFC) was determined using a colorimetric method [51]. Briefly, 0.3 mL of sample solution (0.2 mg/mL) was mixed with 0.95 mL of 60% ethanol and 0.075 mL of 5% NaNO_2_. After 8 min, 0.075 mL of 10% Al (NO_3_)_3_ was added, followed by 1.0 mL of NaOH and 0.1 mL of 60% ethanol. The mixture was allowed to react for 12 min, after which the absorbance was determined at 510 nm. TFC was expressed as rutin equivalent (RE) mg per gram of extract, calculated using the standard curve 7.1444x − 0.00638 (R^2^ = 0.9995).

### 4.7. Antioxidant Capacity of DFH Phenolics in Different Forms

#### 4.7.1. DPPH Free Radical Scavenging Activity

The DPPH radical scavenging assay was conducted following a modified method [52]. Briefly, 40 μL of samples or vitamin C (Vc) (1000, 500, 250, 125, and 62.5 μg/mL) was mixed with 160 μL of 0.1 mM DPPH reagent. The mixture was incubated at room temperature for 30 min in the dark, followed by measuring the absorbance at 517 nm (A_Sample_). A control group containing 20 μL methanol and 180 μL DPPH reagent (A_Control_) was used for baseline correction. All experiments were performed in quintuplicate. The scavenging activity was calculated as following equation:

$$DPPH\;scavenging\;activity\;\left(\%\right)=[(A_{Control}-A_{Sample})/A_{Control}]\times 100$$

#### 4.7.2. ABTS Radical Scavenging Activity

The ABTS radical scavenging assay was performed as described by Luo et al. [53]. Briefly, the ABTS solution was diluted with methanol to an absorbance of 0.70 ± 0.02 at a wavelength of 734 nm. In total, 25 μL samples or Vc (1000, 500, 250, 125, and 62.5 μg/mL) were mixed with 200 μL of ABTS solution. After 6 min, the absorbance was measured at 734 nm (A_Sample_). A control group, replacing the extract with methanol, was used for baseline correction (A_Control_). All experiments were conducted in quintuplicate. The scavenging activity was calculated as follows formula:

$$ABTS\;scavenging\;activity(\%)=[(A_{Control}-A_{Sample})/A_{Control}]\times 100$$

#### 4.7.3. Ferric-Reducing Antioxidant Capacity (FRAP) Assay

The FRAP assay was performed with modifications [54]. The FRAP reagent was prepared by mixing acetate buffer (300 mmol/L, pH 3.6), 2,4,6-Tri(2-pyridyl)-s-triazine (TPTZ) solution (10 mmol/L), and FeCl_3_ (20 mmol/L) in a 10:1:1 ratio (*v*/*v*/*v*). The acetate buffer was formulated by dissolving 0.7750 g of sodium acetate along with 4 mL glacial acetic acid in 250 mL deionized water. For the TPTZ solution, 0.0781 g of TPTZ reagent was dissolved in 25 mL HCl (40 mmol/L concentration). The FeCl_3_ solution was produced by mixing 0.1 g FeCl_3_ crystals with 100 mL purified water. The FRAP reagent was preheated at 30 °C before use. For the assay, 0.5 mL of sample (1000, 500, 250, 125, and 62.5 µg/mL) was mixed with 4.5 mL of preheated FRAP reagent and incubated at 30 °C for 10 min. The absorbance was measured at 593 nm using a microplate reader (SpectraMax M5, Molecular Devices, San Jose, CA, USA). 

### 4.8. Cell Experiments

#### 4.8.1. Estimation of DFH Extracts on HepG2 Cell Viability

HepG2 cells were seeded into a 96-well plate at a density of 1 × 10^5^ cells/mL (200 μL/well) and incubated for 24 h. The cytotoxicity of different DFH extracts (FP-DFH, EP-DFH, BP-DFH, UFP-DFH, UEP-DFH, and UBP-DFH) were evaluated using the MTT assay. The control group received 200 μL of DMEM, while the experimental groups were treated with 200 μL of DFH extracts at concentrations of 50, 100, and 200 μg/mL. After 24 h of incubation, 150 μL of MTT solution (0.5 mg/mL) was added to each well and incubated for an additional 4 h. DMSO was employed to solubilize the formazan crystals prior to absorbance determination at 570 nm using a microplate spectrophotometer.

#### 4.8.2. Determination of Intracellular ROS Content

HepG2 cells were seeded in 6-well plates (1 × 10^5^ cells/mL) and pre-incubated for 12 h until 70–80% confluency was achieved. The cells were divided into four groups as follows: (1) control group: treated with 2 mL DMEM complete medium for 24 h; (2) model group: treated with 1 mL DMEM complete medium for 24 h, and followed by 1 mL of 1 mM H_2_O_2_ for another 24 h; (3) positive control group: treated with 1 mL of 100 μg/mL VC for 24 h, and followed by 1 mL of 1 mM H_2_O_2_ for 24 h; and (4) sample group: treated with 1 mL of 100 μg/mL DFH extracts for 24 h, and followed by 1 mL of 1 mM H_2_O_2_ for 24 h. After treatment, HepG2 cells were washed with cold PBS and incubated with 1 mL of 10 μM DCFH-DA in the dark at 37 °C for 30 min. After that, the cells were washed twice with cold PBS, and intracellular green fluorescence intensity was quantified using flow cytometry [2].

#### 4.8.3. Determining Glutathione (GSH) Pathways in H_2_O_2-_Induced HepG2 Cells

Cell culture and treatment protocols were performed as the aforementioned procedures as Section 4.8.2. The levels of glutathione (GSH) and the enzymatic activities of glutathione synthetase (GS), glutamic acid cysteine ligase (GCL), glutathione reductase (GR), and glutathione peroxidase (GSH-Px) were measured using commercially available assay kits (Nanjing Jiancheng Biotechnology Co., Ltd., Nanjing, China).

#### 4.8.4. Estimating Antioxidant Enzyme Activities

The activities of antioxidant enzymes, including superoxide dismutase (SOD), catalase (CAT), NAD(P)H quinone oxidoreductase 1 (NQO1), and heme oxygenase-1 (HO-1) were measured using commercial assay kits (Nanjing Jiancheng Biotechnology Co., Ltd.) following the manufacturer’s protocols.

### 4.9. Immunofluorescence Analysis of Nrf2 Protein by DAPI Staining

Following fixation in 4% paraformaldehyde for 15 min, cells were permeabilized using 0.1% Triton X-100 (10 min) before being incubated with 5% BSA blocking solution for 1 h. Primary antibody against Nrf2 (1:200 dilution) was added and incubated overnight at 4 °C. Following washes, cells were subjected to a 1 h incubation with fluorophore-conjugated secondary antibodies (diluted 1:500 in PBS) under ambient temperature conditions. Nuclei were counterstained with DAPI (1 μg/mL) for 5 min. Fluorescence images were captured using a confocal microscope.

### 4.10. Molecular Docking Analysis

Molecular docking analysis was conducted to explore the interactions between bioactive compounds (vanillic acid) and the target protein Nrf2. The 3D structures of vanillic acid were retrieved from the PubChem database (https://pubchem.ncbi.nlm.nih.gov/, accessed on 13 October 2024), while the Nrf2 structure (PDB ID: 7JRA) was obtained from the Protein Data Bank (https://www.rcsb.org/, accessed on 20 October 2024). Using PyMol (v2.5.0), water molecules and ligands were removed from the protein structure. Molecular docking was performed using AutoDock 4.2, and the results were visualized to evaluate binding interactions. Binding energy values were calculated to assess the stability and affinity of the ligand–protein complexes.

### 4.11. Data Processing and Statistical Analysis

All assays were conducted with three biological replicates, with results displayed as arithmetic mean ± standard deviation (SD). The data were analyzed using one-way ANOVA, with Tukey’s multiple comparisons (significance threshold set at *p* < 0.05). Principal component analysis (PCA) and additional data processing were performed using Origin 8.5 software (OriginLab, Northampton, MA, USA).

## 5. Conclusions

In this study, the changes in the chemical composition and biological activity of phenolic extracts from DFH before and after the UHP treatment were compared. Among them, 149 differential compounds were identified in the DFH extracts after the UHP treatment, and the contents of TFC and TPC with their oxidation activity were significantly increased. Particularly in the UEP-DFH, the IC_50_ values in ABTS and DPPH were reduced by 49.6% and 64.1%, respectively. Moreover, UHP-treated DFH extracts (especially UFP-DFH and UEP-DFH) promoted the synthesis and metabolism of GSH (GCL, GS, GR, and GSH-px) and activated antioxidant enzymes (SOD, CAT, HO-1, and NQO1) in H_2_O_2_-induced HepG2 cells, which significantly inhibited ROS-induced oxidative stress injury. The potential mechanism may be related to the activated Nrf2 pathway. This study revealed the effects of UHP treatment on the antioxidant activity and mechanism of DFH extracts, emphasizing the significant potential of the UHP technology in improving extraction efficiency and antioxidant activity, providing valuable insights for food or functional food processing.

## Figures and Tables

**Table 1:** Main composition of various DFH extracts using UHPLC-OE-MS in negative ion mode (relative quantitation).

No.	Compound	RT (min)	[M-H]- (*m*/*z*)	Molecular Formula	FP	UFP	EP	UEP	BP	UBP
1	Coniferaldehyde	0.50	177.0557	C_10_H_10_O_3_	0.4995	0.5641	0.7070	0.5064	0.5503	0.6177
2	Vanillin	0.56	151.0404	C_8_H_8_O_3_	1.5430	1.9668	2.3606	1.8610	1.9462	1.5387
3	Gentisaldehyde	0.66	137.024	C_7_H_6_O_3_	0.4955	0.4559	0.5286	0.3846	0.4438	0.4551
4	1,2,3-Trihydroxybenzene	0.83	125.0246	C_6_H_6_O_3_	0.0883	0.1032	0.5325	0.2491	0.8958	0.5310
5	Rhein	1.03	283.0271	C_15_H_8_O_6_	0.0189	0.0044	0.0030	——	0.1026	0.0549
6	Phthalic acid	1.48	165.0195	C_8_H_6_O_4_	——	——	0.5784	0.2747	0.9825	1.0042
7	4-Methylcatechol	1.77	123.0451	C_7_H_8_O_2_	0.1824	0.1571	——	0.2920	0.2259	0.1935
8	p-Anisic acid	1.83	151.0404	C_8_H_8_O_3_	0.5206	0.6708	——	0.6720	0.6950	0.7531
9	2-Hydroxy-2-methylbutyric acid	2.00	117.0557	C_5_H_10_O_3_	26.2968	23.6317	172.1057	170.1849	43.5481	38.4994
10	2,3-Dihydroxybenzoic acid	2.25	153.0194	C7H6O4	1.7250	1.5524	2.8067	1.9010	1.4105	1.0499
11	6-Hydroxynicotinic acid	2.80	138.0196	C_6_H_5_NO_3_	0.0587	0.1927	0.0902	0.1664	0.1137	0.1133
12	2-Isopropylmalic acid	2.97	175.0614	C_7_H_12_O_5_	8.1956	10.0603	62.0692	35.6353	24.2040	21.0959
13	4-Hydroxy-3-methylbenzoic acid	3.00	151.0402	C_8_H_8_O_3_	2.5323	2.6305	2.8736	2.5382	0.7799	1.1939
14	3,5-Dihydroxybenzoic acid	3.14	153.0192	C_7_H_6_O_4_	3.7772	3.6628	11.0047	9.7879	10.5693	7.0524
15	L-Phenylalanine	3.26	164.0717	C_9_H_11_NO_2_	0.0234	0.0415	0.0901	0.0738	——	0.4390
16	D-Pantothenic acid	3.32	218.1035	C_9_H_17_NO_5_	2.3129	2.8695	0.0286	2.1183	0.0402	0.0285
17	Xanthurenic acid	3.52	204.0301	C_10_H_7_NO_4_	0.0095	0.0088	——	1.0110	0.3960	0.1246
18	4-Hydroxycinnamic acid	3.63	163.0404	C_9_H_8_O_3_	0.9138	0.8933	1.3231	0.8071	0.8909	0.6438
19	Aucubin	3.70	405.1406	C_15_H_22_O_9_	0.1438	0.0766	0.3219	0.2886	0.1333	0.0883
20	Trans-Ferulic acid	3.72	193.0506	C_10_H_10_O_4_	0.4977	0.5037	2.2448	2.3360	1.3769	1.0619
21	Kynurenic acid	3.78	188.0357	C_10_H_7_NO_3_	0.2485	1.9866	3.5634	3.5251	0.9928	0.0171
22	Acetylleucine	3.87	172.0979	C_8_H_15_NO_3_	7.2885	8.1930	8.7119	10.6443	2.2280	0.2493
23	Vanillic acid	4.01	167.0348	C_8_H_8_O_4_	120.8814	147.6204	1197.8593	834.2383	533.5137	370.9397
24	Astringin	4.05	405.1187	C_20_H_22_O_9_	0.3206	0.3241	1.4720	1.0375	0.8699	0.4659
25	Suberic acid	4.50	173.0822	C_8_H_14_O_4_	15.2357	16.6105	6.7092	13.5709	71.7242	69.5280
26	Azelaic acid	5.07	187.0977	C_9_H_16_O_4_	175.1019	198.6162	30.8073	117.7309	448.1798	390.5198
27	3-Hydroxycinnamic acid	5.11	163.0404	C_9_H_8_O_3_	3.5035	1.4209	32.2576	21.8638	5.3865	13.8460
28	Oxypurinol	1.69	151.0264	C_5_H_4_N_4_O_2_	0.6179	——	0.3730	1.2113	0.4851	0.5641
29	Traumatic acid	4.47	227.1289	C_12_H_20_O_4_	0.0546	0.0139	——	1.8766	0.2041	0.1923
30	Nepodin	1.27	215.0736	C_13_H_12_O_3_	0.2625	0.3039	0.3475	0.2099	0.1406	0.0642
31	3-Methyluric acid	4.49	227.0467	C_6_H_6_N_4_O_3_	0.3854	0.5446	0.6132	0.5731	0.1803	0.1786
32	Guanosine-5′-monophosphate	6.84	362.0525	C_10_H_14_N_5_O_8_P	1.6239	1.8488	0.0309	1.4545	0.0396	——

## Data Availability

Data will be made available on request.

## Supplementary Materials

The following supporting information can be downloaded at: https://www.mdpi.com/article/10.3390/molecules30081836/s1, Table S1: DFH extracts detected 374 compounds in negative ion mode.; Table S2: 149 Differential compounds in DFH extracts.

## Abbreviations

The following abbreviations are used in this manuscript:

FP (free phenolic fraction), EP (esterified phenolic fraction), BP (bound phenolic fraction), ROS (reactive oxygen species), MDA (malondialdehyde), UHP (ultra-high pressure), DFH (*Dendrobium fimbriata* Hook), TCM (traditional Chinese medicine), UHPLC-HRMS/MS (ultra-high-performance liquid chromatography–high-resolution mass spectrometry), DPPH (2,2-diphenyl-1-picryhydrazyl), ABTS (2,2′-azino-bis (3-ethylbenzothiazoline-6-sulfonic acid)), FRAP (ferric-reducing antioxidant power), VIP (variable importance in projection), partial least square-discriminant analysis (PLS-DA), TPC (total phenolic content), TFC (total flavonoid content), GAE (gallic acid equivalent), RE (rutin equivalent), MTT (3-(4,5-Dimethylthiazol-2-yl)-2,5-diphenyltetrazolium bromide), Vc (vitamin C), GSH (glutathione), GS (glutathione), GCL (glutamate-cysteine ligase), GR (glutathione reductase), GSH-Px (glutathione peroxidase), SOD (superoxide dismutase), CAT (catalase), NQO1 (NAD(P)H quinone oxidoreductase 1), HO-1 (heme oxygenase-1), PCA (principal component analysis), Keap1 (Kelch-like ECH associated protein 1), ARE (antioxidant response element), DCFH-DA (2′,7′-dichlorofluorescin diacetate), FBS (Fetal bovine serum), DMEM (Dulbecco’s modified Eagle’s medium), HepG2 (human hepatocellular carcinoma cell line).
